# Curcumin Inhibits Gastric Inflammation Induced by *Helicobacter Pylori* Infection in a Mouse Model

**DOI:** 10.3390/nu7010306

**Published:** 2015-01-06

**Authors:** António M. Santos, Teresa Lopes, Mónica Oleastro, Inês Vale Gato, Pauline Floch, Lucie Benejat, Paula Chaves, Teresa Pereira, Elsa Seixas, Jorge Machado, António S. Guerreiro

**Affiliations:** 1 Serviço de Medicina 4—Hospital de Santa Marta/Centro Hospitalar de Lisboa Central, Rua de Santa Marta, 50, 1169-024 Lisboa, Portugal; E-Mail: antonioguerreiro@hotmail.com; 2 CEDOC—Nova Medical School—Faculdade de Ciências Médicas Campo Mártires da Pátria 130, 1169-056 Lisboa, Portugal; E-Mails: maria.lopes@fcm.unl.pt (T.L.); eseixas@igc.gulbenkian.pt (E.S.); 3 Departamento de Doenças Infecciosas, Instituto Nacional de Saúde Dr. Ricardo Jorge, Avenida Padre Cruz, 1649-016 Lisboa, Portugal; E-Mails: monica.oleastro@insa.min-saude.pt (M.O.); ivaledegato@gmail.com (I.V.G.); jorge.machado@insa.min-saude.pt (J.M.); 4 Bacteriology Laboratory, Bordeaux University, 146 rue Léo Saignat F-33000 Bordeaux, France; E-Mails: pauline-floch@hotmail.fr (P.F.); lucie.benejat@u-bordeaux2.fr (L.B.); 5 Serviço de Anatomia Patológica—Instituto Português de Oncologia Dr. Francisco Gentil, R. Prof. Lima Basto, 1099-023 Lisboa, Portugal; E-Mails: pchaves@ipolisboa.min-saude.pt (P.C.); tpereira@ipolisboa.min-saude.pt (T.P.)

**Keywords:** *H. pylori*, curcumin, nutritional approach, secondary prevention, mouse model

## Abstract

*Helicobacter pylori* (*H. pylori*) infection triggers a sequence of gastric alterations starting with an inflammation of the gastric mucosa that, in some cases, evolves to gastric cancer. Efficient vaccination has not been achieved, thus it is essential to find alternative therapies, particularly in the nutritional field. The current study evaluated whether curcumin could attenuate inflammation of the gastric mucosa due to *H. pylori* infection. Twenty-eight C57BL/6 mice, were inoculated with the *H. pylori* SS1 strain; ten non-infected mice were used as controls. *H. pylori* infection in live mice was followed-up using a modified ^13^C-Urea Breath Test (^13^C-UBT) and quantitative real-time polymerase chain reaction (PCR). Histologically confirmed, gastritis was observed in 42% of infected non-treated mice at both 6 and 18 weeks post-infection. These mice showed an up-regulation of the expression of inflammatory cytokines and chemokines, as well as of toll-like receptors (TLRs) and MyD88, at both time points. Treatment with curcumin decreased the expression of all these mediators. No inflammation was observed by histology in this group. Curcumin treatment exerted a significant anti-inflammatory effect in *H. pylori*-infected mucosa, pointing to the promising role of a nutritional approach in the prevention of *H. pylori* induced deleterious inflammation while the eradication or prevention of colonization by effective vaccine is not available.

## 1. Introduction

*Helicobacter pylori* (*H. pylori*) is one of the most common human pathogens since it infects the gastric mucosa of about 50% of the world’s population [1]. The majority of infections are asymptomatic, acquired by an overwhelming majority of the pediatric population, making the infection life-long without effective bacterial eradication. Additionally, epidemiological studies associating the infection with a higher risk of gastric malignancy lead the World Health Organization for Research in Cancer to classify *H. pylori* as a class I *carcinogen* [2]. *H. pylori* infection is *a major r*isk factor for gastric cancer development because it triggers a stepwise sequence in the gastric mucosa starting with superficial gastritis, which can progress to chronic gastritis, atrophic gastritis, intestinal metaplasia, dysplasia, and, ultimately, gastric carcinoma [3]. The bacteria induce a host immune response (innate and adaptative), but the persistence of the infection suggests that the response is not effective in eliminating the infection. Furthermore multiple lines of evidence suggest that the immune response contributes to the pathogenesis associated with the infection.

Over the last two decades, several experimental models of *H. pylori* infection have been developed to investigate the pathogenesis of this infection. Using a mouse-adapted *H. pylori* strain (Sidney Strain, SS1), Lee *et al.* established a model of long-term and high bacterial colonization in mice [4]. Although *H. pylori* is known to be non-invasive, an extensive inflammatory reaction is provoked in the gastric mucosa as a consequence of the infection [5]. This reaction is characterized by a mucosal infiltration of inflammatory cells, especially neutrophils, which is mediated by enhanced expression of proinflammatory chemokines and cytokines [6,7].

Given the high prevalence of *H. pylori* infection worldwide, the high costs of antibiotic treatment, and the increasing rates of antibiotic resistance, considerable efforts have been made to develop vaccines against *H. pylori*. However, efficient vaccination has not been achieved in human beings to date [8]. Thus it is essential to find alternative therapies, particularly related to nutrition, such as plant extracts that contain numerous polyphenols, which have been shown to reduce inflammation [9]. Curcumin, a naturally occurring phytochemical and an extract of *Curcuma longa* (turmeric), is known to possess many pharmacologic properties and is widely used in herbal medicine, such as for peptic diseases in ayurvedic medicine [10]. Moreover, curcumin has proven to exhibit remarkable anticarcinogenic, anti-inflammatory, and antioxidant properties [11,12].

Thus, curcumin may be a potential agent for controlling inflammation associated with *H. pylori* infection. The aim of the present study is to evaluate the anti-inflammatory effect of curcumin using the experimental model of *H. pylori* chronic infection.

## 2. Experimental Section

### 2.1. Experimental Infections

A total of 38 pathogen-free male C57BL/6 5-week-old mice (Harlan Laboratories, Castellar, Spain—Genetic code: C57BL/6JOla-Hsd) were used in compliance with the national animal guidelines. The present study was specifically approved by the National Animal Care Committee from the Portuguese General Veterinary Direction.

The animal facility had a regulated room temperature (21 °C–24 °C), humidity (55% ± 10%), and an artificial standard 12-h light/12-h dark cycle.

All mice had *ad libitum* access to tap water and feeding (standard diet—Mmucedola srl-Italy-4RF21 certificate batch: 250202) and were kept in standard mice plastic cages (size 13 cm high, 30 cm length and 20 cm wide) with a Souralit 3000 beeding (batch: 118/11 with no pre-treatment).

After an acclimatization period of two weeks, the mice were divided into three different groups: Control group (CG), *n* = 10, Infected group (IG), *n* = 14, Infected group treated with Curcumin (IG + C), *n* = 14. Using a 20-gauge ballpoint metal feeding tube (Harvard Apparatus, Inc., Holliston, MA, USA), mice were inoculated intragastrically, on three consecutive days, with either 0.1 mL of *H. pylori* SS1 cell suspension containing 10^8^ colony-forming units/mL (IG and IG + C) or phosphate buffered saline (PBS) (CG). Two weeks after infection, the IG + C group received 0.5 mL of a lipidic solution of curcumin (Sigma-Aldrich, Sintra, Portugal) (500 mg/kg) [13,14], and the IG received PBS; both regimens were given three times a week, for 6 and 18 weeks by gavage.

At Week 6, the five non-infected mice (CG) and seven mice from both the IG and IG + C groups, were killed. The remaining mice of each group were killed at Week 18.

The mice were food-deprived for 14 h prior to euthanization by cervical dislocation. For each mouse, half of the stomach was totally processed for histology and immunohistochemistry, and the remaining half was split in two, one part was immediately conserved at −80 °C in RTL buffer (Qiagen GmbH, Hilden, Germany) with 2-mercaptoethanol (Sigma-Aldrich, Sintra, Portugal) for RNA extraction, and the remaining half was used for DNA extraction.

### 2.2. Urea Breath Test (^13^C-UBT) and Quantitative Real-Time PCR (qPCR) for H. Pylori

The presence or absence of active *H. pylori* infection was evaluated for all mice one week after the infection, and again at both 6 and 18 weeks after infection, before euthanasia, with an adapted ^13^C-UBT, as previously described [15].

A qPCR was performed on extracted DNA from the stomach in order to evaluate the putative effect of curcumin treatment on *H. pylori*. 

A real-time PCR based on SYBR Green technology was used to quantify *H. pylori* and mouse GAPDH (Glyceraldhehyde 3-phosphate deshydrogenase), an epithelial cell target, in stomach DNA extracts. *H. pylori* specific PCR targets the gene encoding 23S rRNA of *H. pylori* as already described [16]. GAPDH primers (mGapdh1for: CTGCAGGTTCTCCACACCTATG; mGapdh1rev: GAATTTGCCGTGAGTGGAGTC) were designed using Primer Express software (Life Technologies). Each sample was tested twice for 23S rRNA and GAPDH (Eurofins, Luxembourg, Luxembourg).

Two standard ranges were prepared, the first one with DNA extract from bacterial calibrated suspension (UFC/mL) of *H. pylori* SS1 strain, the second with DNA from murine epithelial cells (m-Iccl2 line) [17] at a known concentration. Two standard curves were obtained and used to quantify the number of bacteria and murine cells, respectively, by μL of DNA. Final results were expressed by the ratio bacteria/μL of DNA on murine cells/μL of DNA.

LightCycler(r) 480 SYBR(r) Green I Master Mix (Roche Diagnotics reagent was used according to supplier’s recommendations. PCR were performed on Light cycler 480 (Roche Diagnostics, Bâle, Switzerland). Following initial denaturation at 95 °C for 10 min, 45 amplification cycles (95 °C for 10 s, 60 °C for 10 s, and 72 °C for 15 s) were performed. Fluorescence was measured at 640 nm after each cycle. This was followed by a melting program of 95 °C for 60 s and 38 °C for 50 s at a temperature transition rate of 20 °C/s and 90 °C for 0 s (hold time) at a rate of 0.1 °C/s, with continuous monitoring of the fluorescence. The final step consisted of cooling at 2.2 °C/s to 40 °C. The results are presented as the ratio 23S rRNA/GAPDH.

### 2.3. Immunohistochemistry and Histology

Sections of buffered formalin-fixed paraffin-embedded tissue blocks (2 μm thick) were cut onto Superfrost plus slides. After baking in an oven, the 2 μm sections were de-waxed, rehydrated, and subjected to epitope antigen retrieval (20 min, 94 °C) with Target Retrieval Solution High pH 50× EnvisionTM Flex (Dako, Glostrup, Denmark) in a pre-treatment module PTlink (Dako). Endogenous peroxidase was blocked with 2% H_2_O_2_ in absolute methanol for 10 min. Immunostaining was performed by the peroxidase-indirect-polymer method. Primary polyclonal rabbit anti-*Helicobacter Pylori* (Dako, Glostrup, Denmark), was incubated for 30 minutes at room temperature. The labeled polymer HRP anti-rabbit (Dako EnVisionTM, Carpinteria, USA) detection staining system was used at room temperature for 30 minutes and DAB (3,3′-diaminobenzidine) for visualization. Sections were counterstained with Mayer’s Hematoxylin. As positive control a mouse gastric specimen previously known to be positive was used for HP. For negative control, primary antibody was omitted during the staining.

For histological analysis, mouse stomach samples were fixed with 10% buffered formalin solution, pH 7.0, routinely processed, and embedded in paraffin. Sections of tissue blocks 4 μm thick were cut onto glass slides and stained with hematoxylin-eosin (H & E). Inflammation was blindly observed and graded from zero to three as described elsewhere [18].

### 2.4. Real-Time PCR Arrays

For PCR arrays, three mice from each group, CG, IG, and IG + C, at all time-points of the experiment, were randomly chosen and tested individually. Total RNA from mouse stomach samples was extracted using the RNeasy Mini kit (Qiagen GMbH, Hilden, Germany), including the DNase I digestion step to eliminate residual genomic DNA, as recommended by the manufacturer. Integrity and concentration of each RNA sample was analyzed in the Agilent 2100 Bioanalyzer. Then 500 ng of RNA were reverse transcribed to single-stranded cDNA using the RT^2^ First Strand kit (Qiagen) according to manufacturers’ protocols.

Analysis of expression of 84 inflammation mediator genes, as well as of five housekeeping genes (HKG), was performed by PCR array, using the RT^2^ Profiler PCR Array mouse inflammatory response and immunity pathway (SABioscience, Qiagen), in a 384-well format, using the Applied Biosystems 7900HT Fast Real-Time PCR System. Data was normalized to the mean values of the five HKG, and the relative amount of RNA was calculated using the 2^−ΔCt^ method.

Fold-change calculations were done using SABiosciences’ data analysis software, which automatically calculates the fold-change in gene expression between the infected non-treated mice and the control group (IG *versus* CG), and between the infected and curcumin treated mice and the control group (IG + C *versus* CG). Fold-change values greater than one indicate an up-regulation, while fold-changes less than one indicate a down-regulation.

### 2.5. Statistical Analysis

Differences on the histology score were tested by the Mann-Whitney U test. Student’s *t* test was used for the remaining statistics. Both tests were considered as statistically significant when *p* < 0.05. Results are expressed as averages ± standard deviation (SD) of *n* observations.

## 3. Results

### 3.1. H. Pylori Status

**Figure 1:**
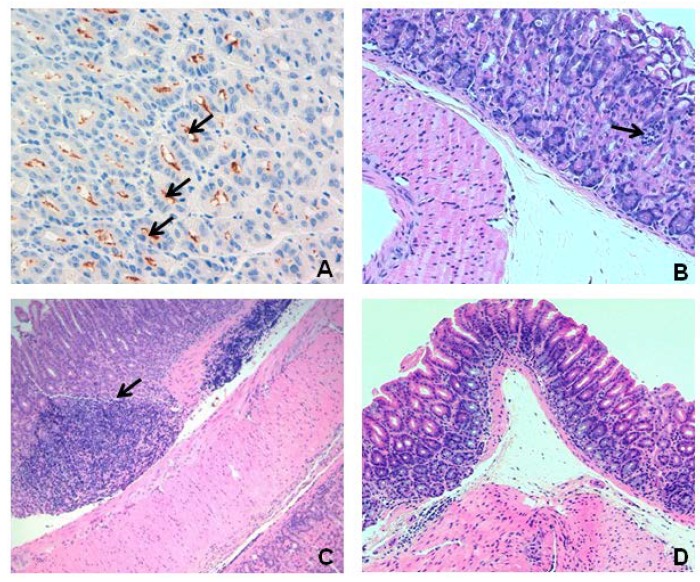
Gastric mucosa of an infected mouse. Immunohistochemistry for *H. pylori*, 40× (black arrows indicate bacteria) (**A**). Mucosal inflammation at 6 (**B**) and 18 weeks (**C**) of the infected non-treated mice (hematoxylin & Eosin (H & E) × 10). Black arrows indicate small lymphoid aggregates at the mucosa (**B**) and a well-defined lymphoyid aggregate at the submucosa (**C**)—Normal gastric mucosa (H & E × 10) of infected mice treated with curcumin at 18 weeks (**D**).

At all the time points analyzed, 1 week, 6 and 18 weeks after the infection, *H. pylori* was detected in all the 28 inoculated mice, both by ^13^C-UBT (Supplementary Figure S1) and by immunohistochemistry (Figure 1A), independent of treatment with curcumin.

**Figure 2:**
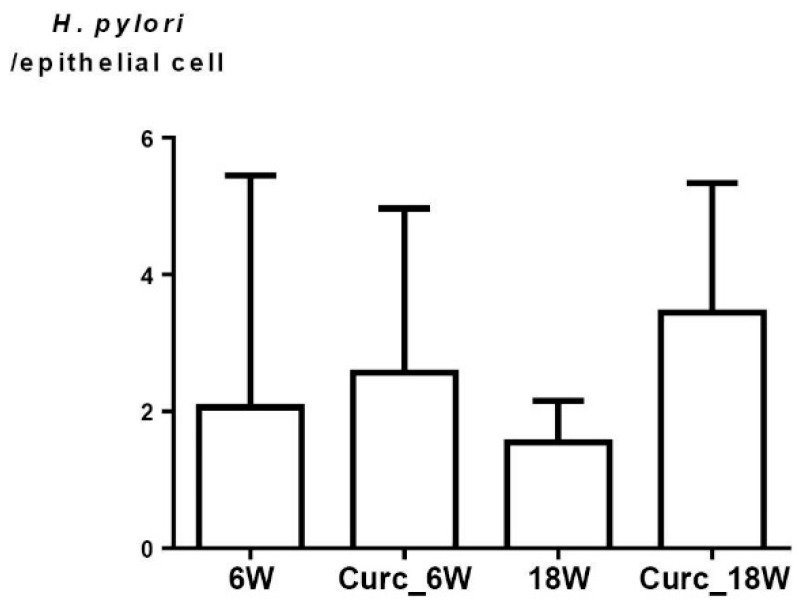
Quantitative real-time polymerase chain reaction (PCR) evaluating the load of *H. pylori* in the mouse gastric mucosa. Values denote the means of the ratio 23S rRNA/GAPDH, obtained for each of the mice analyzed in each group, each mouse tested in duplicate. 6W and 18W, refer to the groups of infected mice at 6 and 18 weeks, respectively; Curc_6W and Curc_18W, refer to the groups of infected mice, receiving treatment with curcumin, at 6 and 18 weeks, respectively.

Mice from the CG were all negative for *H. pylori* infection. Interestingly, from week 1 to week 18, a non-significant reduction in the ^13^CO_2_ values assessed by ^13^C-UBT was observed for all the infected mice. These results were confirmed by qPCR, for which no *H. pylori* DNA was detected among the non-infected mice, while no difference in *H. pylori* DNA content was observed between infected mice at 6 and 18 weeks, either treated or non-treated with curcumin (Figure 2).

### 3.2. Histology

The inflammation of gastric mucosa was analyzed without previous knowledge by the observer. For the CG mice, no inflammation of gastric mucosa was observed at either point in time. Among the infected mice, three of seven (42.8%) presented moderate inflammation (Score 2) at Week 6, and mild inflammation (Score 1) was also observed in three out of seven mice (42.8%) at 18 weeks (Figure 1B,C and Supplementary Table S1). No inflammation of gastric mucosa was observed in the curcumin-treated mice at either 6 or 18 weeks post-infection (Figure 1D). Differences among infected treated and non-treated groups were statistically significant for the 6 weeks of infection (U = 3.5, *p* = 0.004), but not for the 18 weeks of infection (U = 14.0, *p* = 0.209) (Supplementary Table S1).

### 3.3. Real-Time PCR Arrays

**Figure 3:**
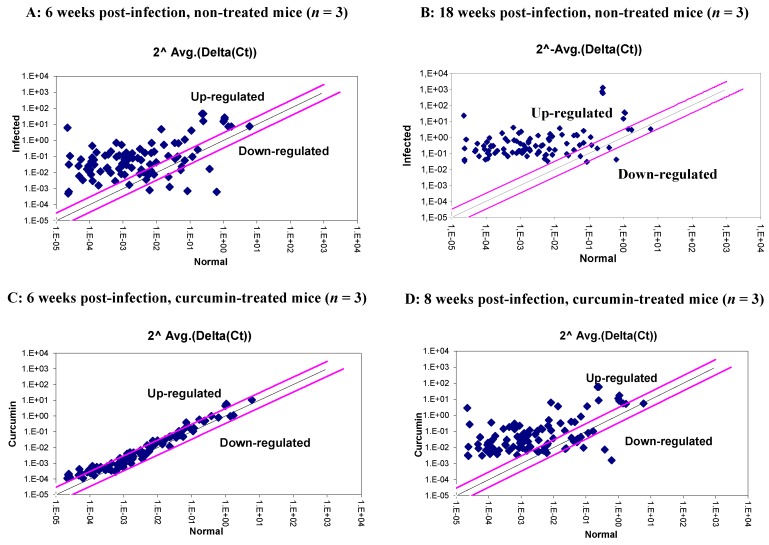
Relative expression level (2^−ΔCt^) for the 84 mouse inflammatory response and immunity encoding genes, between normal mice and *Helicobacter pylori-*infected mice at Week 6 post-infection (**A**) and at Week 18 post-infection (**B**); and between normal mice and *Helicobacter pylori*-infected mice treated with curcumin at Week 6 post-infection (**C**) and at Week 18 post-infection (**D**). Values denote the means obtained for each of the mice analyzed in each group, each mouse tested in duplicate (± SD). The grey lines indicate a three-fold change in gene expression threshold.

Of the 84 mouse inflammatory response and immunity pathway genes analyzed, 69 showed at least a three-fold difference in gene expression between normal mice (CG) and the infected non-treated mice (IG). At week 6, up-regulation was observed in 64 genes, while five genes appeared to be down-regulated in the infected mice, (Figure 3A and Supplementary Table S2). At week 18, up-regulation was observed in 78 genes, and only two genes appeared to be down-regulated in the infected mice (Figure 3B and Supplementary Table S2).

**Figure 4:**
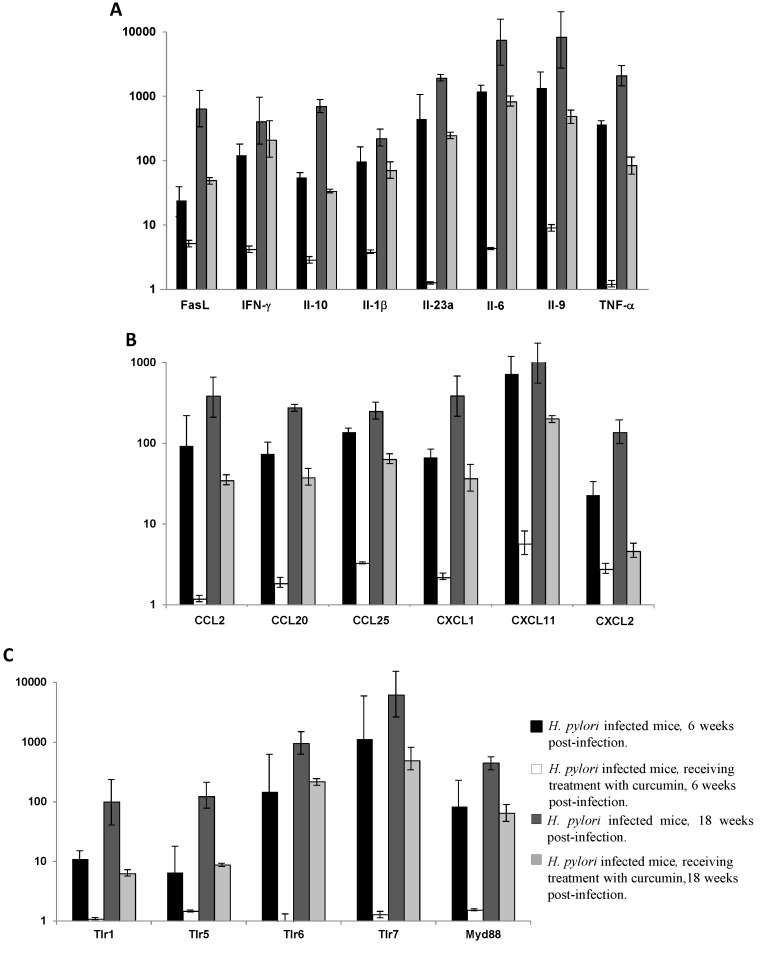
Fold-change in expression of mouse inflammatory response and immunity encoding genes, as determined by PCR arrays and calculated using the 2^−ΔCt^ method, comparing the *Helicobacter pylori*-infected non-treated mice and the infected and treated with curcumin mice *versus* non-infected mice, at Week 6 post-infection and at Week 18 post-infection: (**A**) mouse cytokines; (**B**) mouse chemokines; (**C**) mouse toll-like receptors (TLR) and MyD88. Values denote the means obtained for each of the mice analyzed in each group, each mouse was tested in duplicate (± SD). *p* < 0.05 for all the comparisons between the infected non-treated and the infected curcumin treated groups except for IFN-γ at Week 18. Values denote the means obtained for each of the three mice analyzed in each group, each mouse was tested in duplicate (± SD).

The treatment of mice with curcumin for 6 and 18 weeks down-regulates the expression of almost all studied genes. This effect was more pronounced at 6 weeks of treatment than at 18 weeks (Figure 3C,D and Supplementary Table S2). The pro-inflammatory cytokines and receptors were included in the group of up-regulated genes, at Week 6, among which were the main genes involved in the immune response against *H. pylori* infection: IL-1β, IL-6, IL-9, IL-10, IL-23a, IFN-γ, TNF‑α, and Fasl (range of fold-change between infected and non-infected mice varied from 23.9 to 1338.6) (Figure 4A).

The same scenario was observed for the chemokines, with special emphasis on CCl2, CCL20, CCL25, CxCL1, CxCL2, and CxCL11, with fold-change ranging from 22.7 to 717.5 (Figure 4B).

Among the toll-like receptors (TLRs), the genes encoding for TLR1, TLR5, TLR6, and TLR7 showed the largest increase range in the infected mice (range of fold-change from 6 to 1123), as well as the universal adapter protein MyD88 (fold-change of 83.3), which is used by almost all TLRs to activate the transcription factor nuclear factor kappa beta (NF-κB) (Figure 4C).

At week 18, up-regulation of the genes was even more pronounced, with fold-change ranging between 400.4 and 8251.1 for the main pro-inflammatory cytokines (Figure 4A), and between 135.5 and 1191.9 for chemokines (Figure 4B). Regarding the TLRs, a marked up-regulation was also observed, with fold-change ranging from 99.2 to 6164.5, and a fold-change of 446.3 for the MyD88 encoding gene (Figure 4C).

An increased in expression of the inducible nitric oxide synthetase (iNOS) was also observed for the infected non-treated mice compared to the control group, at both Week 6 (fold-change of 224) and Week 18 (fold-change of 2161).

Treatment of infected mice with curcumin drastically decreased the expression of all the inflammatory mediators, restoring their levels to those similar to the non-infected mice (Figure 3C,D). Indeed, at Week 6, among the 84 inflammatory response and immunity pathway tested genes, only 18 genes were still more expressed in the treated group than in the control group, with fold-changes varying from 3.2 to 9.0. In comparison with the infected non-treated mice, the levels of fold-change in gene expression for the curcumin-treated mice were significantly lower, averaging 100 times less for the cytokines, 70 times less for the chemokines’ group, and 230 times less for the TLRs and MyD88 encoding genes (Figure 4A–C).

At Week 18, 65 out of the 84 genes tested in the curcumin-treated mice were still up-regulated compared to the control group; however the levels of fold-change decreased on average five times when compared to the fold-change observed in the infected non-treated mice (Figure 4A–C). For each gene category the effect of curcumin on the infected mice, compared to the infected non-treated group, was as follows: eleven times decrease in fold-change for the cytokines, seven times for chemokines, and ten times for TLRs and MyD88.

Overall, the effect of curcumin on inflammation due to *H. pylori* infection was more pronounced after 6 weeks of infection than after 18 weeks, even if, in this case, the treatment was more prolonged. Curcumin had a pronounced effect on all inflammatory mediators’ encoding genes, with special emphasis on cytokines, chemokines, and TLRs.

## 4. Discussion

This study focused on the evaluation of the anti-inflammatory properties of curcumin by both histological and molecular approaches, in the context of the chronic infection by *H. pylori*, using the *in vivo* mouse model.

Our data confirm that the mouse-adapted *H. pylori* SS1 strain is well adapted to the mouse stomach milieu, which explains the high infection rate (100%) achieved in this study, even after 18 weeks of infection. Despite the low number of tested mice, the histological analysis performed showed that curcumin was effective in reducing the inflammation of the gastric mucosa of *H. pylori*-infected mice, which was confirmed at the molecular level. At this level the magnitude of the difference in the expression of the inflammatory mediators’ encoding genes between the infected curcumin-treated mice and the infected but non-treated ones strongly supports the powerful anti-inflammatory properties of curcumin.

Inhibition of *H. pylori* growth was unlikely to be a mechanism that contributed to the effect of curcumin observed in this study, since positive results regarding the presence of bacteria were still obtained from both ^13^C-UBT and qPCR. Although the amount of recovered ^13^CO_2_ from breath analysis of all infected groups slightly decreased with time during the experiment, it was not significantly different between the groups at all time points, which was corroborated by the qPCR results. Moreover, the *H. pylori* colonization density measured by immunohistochemistry showed a positive result in all mice including the treated ones.

One can speculate that curcumin, although not bactericidal, may affect the expression/amounts of virulent genes.

Recently, Sintara *et al.* [19] in rats using histology and Di Mario *et al.* [20] in humans, using serology, demonstrated that treatment with curcumin significantly improved gastric inflammation associated with *H. pylori* infection despite persistence of the bacterium, supporting the findings of this study. However, these data are not confirmed by Kundu *et al.* [21] who suggested that curcumin acted two ways during protection against *H. pylori* infection, *i.e.* by eradicating *H. pylori* as well as potentially targeting key molecules involved in the *H. pylori*-induced gastric diseases.

According to Goel *et al.* [22], eradication by curcumin may be dependent on a high dose of this chemopreventive agent, the safety of which still has to be confirmed in animals and humans.

There are two main mechanisms by which *H. pylori* (or its products) may produce gastric inflammation. Firstly, the organism may interact with surface epithelial cells, producing either direct cell damage or the liberation of epithelial-derived pro-inflammatory mediators (chemokines). The epithelial chemokine response may be particularly important in the early stages of *H. pylori*-induced inflammation, with the epithelium acting as a crucial first line of defense against microbial infection. Secondly, *H. pylori*-derived products may gain access to the underlying mucosa, thereby directly stimulating host non-specific and specific immune responses involving the liberation of a variety of cytokine messengers [23].

Our data clearly show an increased expression of a set of chemokines (CCL20, CCL5, CXCL1, CXCL10, CXCL11, CCL25) normally involved in *H. pylori* gastritis [24,25,26] at both 6 and 18 weeks of infection in mice, which decreased after curcumin treatment.

Still in the context of non-specific (innate) immunity, the literature describes an increase in the secretion of TNF-α, IL-6, and IL-1β during *H. pylori* infection, with all three polypeptides being predominantly macrophage-derived cytokines with a wide range of pro-inflammatory actions involved in leukocyte recruitment and activation [27,28]. Our study confirmed these data, showing a marked increase in the expression of these cytokines in the infected mice, which decreased significantly after treatment with curcumin.

Innate immune activation depends on the input of multiple microbial stimuli. Similar to other bacteria, *H. pylori* activates several TLRs on epithelial and dendritic cells (DCs). It is possible that different TLRs ligands derived from the same microbe induce distinct and opposite effects on DCs [29,30,31]. To date, eleven members of the TLRs family have been found to be expressed in mice [32]. TLRs are key molecules mediating the interaction between *H. pylori* and DCs, which is largely dependent on the adaptor protein MyD88 signaling that is used by all TLRs except TLR3 [8]. Our data are consistent with the ones mentioned above, showing a significant increase in MyD88 expression in infected mice, as well as of several TLRs [33]. Once again, treatment of infected mice with curcumin restored the normal range of all these molecules.

With regard to adaptive immunity, the discovery of Th17 cells was a major step in our understanding of CD4 + T cell responses to *H. pylori* infection. The early events in the immune response of immunized and challenged mice include the recruitment of T cells. In murine systems, Th17 cell differentiation depends on the presence of IL-6, transforming growth factor beta (TGF-β1), and CCL20, which are significantly increased in *H. pylori*-stimulated macrophages. Subsequently, IL-23 that is increased in patients with *H. pylori* gastritis will promote Th17 cell proliferation via the STAT3 pathway [33,34]. The NF-κB pathway participates in the production of these inflammatory mediators in response to *H. pylori* [35]. In a similar way to that found with the previously described chemokines, the expression of this set of cytokines was also increased in our infected mice (TGF-β was not measured) and decreased significantly after treatment with curcumin. It is worth noting that gastric epithelial cells stimulated by IL-17 also lead to production of chemokines, including CCL25, chemotactic factor for macrophages, activated monocytes, and DCs. In our study, we found an increase in the expression of this cytokine, probably reflecting IL-17 stimulation, the level of which returned to normal after curcumin therapy.

Shi *et al.* [36] suggest that *H. pylori* infection induces a mixed Th17/Th1 cell response and the Th17/IL-17 pathway modulates Th1 cell responses and contributes to pathology. It is generally accepted that *H. pylori* infection results in a Th1-dominant response and gastric inflammation that depends on Th1 cells is characterized by the production of IFN-γ [37,38,39,40]. Accordingly, the infected mice in this study showed a strong Th1 response, with high expression of IFN-γ at both 6 and 18 weeks after infection, which regressed after treatment with curcumin mainly at Week 6 post-infection.

We may wonder what the rational for the beneficial effects of curcumin is in the context of gastric infection by *H. pylori*. The pathogenesis of this infection is associated with bacterial virulence factors that can induce the activation of NF-κB in gastric epithelial cells [19,41]. This transcription factor is an important regulator of many cellular processes, including the control of immune response and inflammation [42,43]. For example, the NF-κB pathway participates in the production of inflammatory mediators in *H. pylori*-stimulated macrophages critical for differentiation and proliferation of Th17 cells [35]. Curcumin has many biological attributes, including anti-inflammatory properties [19], and most of these effects can be explained by the efficient inhibition of NF-κB mediated by this substance [44,45,46]. Previous studies have shown that curcumin can inhibit NF-κB activation in *H. pylori*-infected gastric epithelial cells [47], as well as in rats [48]. In this last study, *H. pylori*-induced gastric inflammation was associated with increased NF-κB activation and macromolecular leakage, which was reduced by curcumin supplementation. In our study the quantification of NF-κB by an alternative method (like qPCR or immunohistochemistry) was not performed, which is a clear limitation of the study. Identifying the localization of NF-κB in the gastric mucosa by immunohistochemistry, if performed, would also have been of informative relevance.

Another point that deserves attention is the observation that despite curcumin fully abolishing the gastric mucosa inflammation, a total remission of gene expression of inflammation markers was not observed. In our study, we also observed a discrepancy between histological inflammatory scores and inflammatory genes by PCR arrays for all infected mice, before or after curcumin treatment, and the more pronounced gene expression observed at Week 18 post-infection in the non-treated mice had no parallelism in higher histological inflammation scores, compared to Week 6 post-infection. One possible explanation for this is the fact that some adaptive host factors may also contribute to mucosal protection. Considering the limited number of mice in each group and that we did not observe a consistent exuberant mucosal inflammation for any mouse at any time of the experiment, it can be speculated that mouse infection with the human pathogen *H. pylori* might not be the ideal model of inflammation, at least regarding histological parameters.

## 5. Conclusions

In conclusion, as far as we know, this is one of the few studies using both histological and molecular approaches, showing the important anti-inflammatory role of curcumin in the context of chronic *H. pylori* infection. Taking into account the extensive consumption of polyphenols in the human diet (curcumin and others), our data points to the promising role of a nutritional approach in the control of *H. pylori* induced deleterious inflammation while an effective vaccine is not available.

## Supplementary Files

Supplementary File 1
