# Supplementary material for: Curcumin Inhibits Gastric Inflammation Induced by Helicobacter Pylori Infection in a Mouse Model
Source: Nutrients. 2015 Jan 6;7(1):306–20. doi: 10.3390/nu7010306 (PMC4303841; doi:10.3390/nu7010306)
Supplement: Supplementary File 1 [file nutrients-07-00306-s001.docx]

Supplementary information

**Figure S1.** Mean ^13^CO_2_ excess excreted in the mice breath; control group (CG); infected group (IG); infected group treated with curcumin (IG + C), one (1W), six (6W)
and eighteen (18W) weeks after *Helicobacter pylori* infection. Results are expressed
in ‰δPDB.

**Table S1.** Histology inflammation score of mice gastric mucosa.

| Mice | **Week 6** | **Week 18** |
| --- | --- | --- |
| CG-1 * | 0 |  |
| CG-2* | 0 |  |
| CG-3 | 0 |  |
| CG-4 * | 0 |  |
| CG-5 | 0 |  |
| CG-6 |  | 0 |
| CG-7 * |  | 0 |
| CG-8* |  | 0 |
| CG-9 |  | 0 |
| CG-10 * | Δ | 0 |
| IG-1 | 2 |  |
| IG-2 * | 1 |  |
| IG-3 * | 1 |  |
| IG-4 | 2 |  |
| IG-5 | 2 |  |
| IG-6 * | 0 |  |
| IG-7 | 1 | ΔΔ |
| IG-8 * |  | 1 |
| IG-9 |  | 0 |
| IG-10 * |  | 1 |
| IG-11 |  | 1 |
| IG-12 |  | 1 |
| IG-13 * |  | 0 |
| IG-14 | Δ | 0 |

**Table S1.** *Cont.*

| IG + C-1 | 0 |  |
| --- | --- | --- |
| IG + C-2 * | 0 |  |
| IG + C-3 | 0 |  |
| IG + C-4* | 0 |  |
| IG + C-5* | 0 |  |
| IG + C-6 | 0 |  |
| IG + C-7 | 0 | Δ Δ |
| IG + C-8 |  | 0 |
| IG + C-9* |  | 0 |
| IG + C-10 |  | 0 |
| IG + C-11 * |  | 0 |
| IG + C-12 |  | 0 |
| IG + C-13 |  | 0 |
| IG + C-14 * |  | 0 |

Mann-Whitney U test; Δ: U = 3.5, *p* = 0.004; ΔΔ: U = 14.0; *p* = 0.209; *: Mice randomly chosen for PCR Arrays; CG: Control Group; IG: Infected Group; IG + C: Infected group treated with curcumin; Inflammation score graded from 0 to 3: 0—no inflammation, 1—mild inflammation, 2—moderate inflammation, 3—
strong inflammation.

**Table S2.** Fold-change in expression of mouse inflammatory response and immunity encoding genes, as determined by PCR Array using the RT2 Profiler PCR Array mouse inflammatory response and immunity pathway. Significant differences using the Student’s *t* test are marked
in bold.

| **Gene** | **6 weeks after Helicobacter pylori infection** | | | | **18 weeks after Helicobacter pylori infection** | | | |
| --- | --- | --- | --- | --- | --- | --- | --- | --- |
|  | **Fold change  Infected versus Normal** | ***P* value** | **Fold change Infected and  treated with curcumin  versus Normal** | ***P* value** | **Fold change  Infected versus Normal** | ***P* value** | **Fold change Infected and  treated with curcumin  versus Normal** | ***P* value** |
| Bcl6 | 5.10 | 0.19336 | 2.13 | 0.09149 | 126.89 | 0.21506 | 2.24 | 0.28019 |
| C3 | 19.59 | **0.00088** | 1.24 | 0.68634 | 34.76 | **0.04337** | 11.50 | 0.10146 |
| C3ar1 | 16.75 | **0.00047** | 1.34 | 0.55966 | 33.94 | 0.23889 | 8.37 | 0.10175 |
| C4b | 4.25 | **0.04476** | 0.56 | 0.40406 | 36.14 | 0.22945 | 1.61 | 0.32590 |
| Ccl1 | 392.02 | **0.01093** | 1.30 | 0.74913 | 5945.75 | 0.25776 | 503.93 | **0.02173** |
| Ccl11 | 4.74 | 0.06248 | 2.14 | 0.16285 | 16.97 | 0.25203 | 5.02 | **0.01821** |
| Ccl12 | 39.28 | **0.04778** | 2.49 | 0.23965 | 76.26 | 0.28033 | 5.80 | 0.16665 |
| Ccl17 | 56.12 | 0.11945 | 2.00 | 0.36880 | 547.11 | 0.28282 | 48.34 | 0.10068 |
| Ccl19 | 24.02 | 0.16310 | 2.06 | 0.77880 | 92.31 | 0.28559 | 33.36 | 0.16952 |
| Ccl2 | 92.61 | 0.37914 | 1.17 | **0.00956** | 382.71 | 0.36599 | 34.34 | **0.02623** |
| Ccl20 | 73.73 | 0.23523 | 1.82 | **0.03177** | 273.75 | 0.05112 | 37.38 | 0.18513 |
| Ccl22 | 36.52 | 0.22688 | 0.70 | 0.69972 | 144.92 | 0.25425 | 20.90 | 0.19729 |
| Ccl24 | 537.98 | 0.15761 | 3.69 | 0.07793 | 2676.86 | 0.28084 | 351.98 | 0.13169 |
| Ccl25 | 137.41 | **0.04362** | 3.25 | **0.00051** | 247.42 | 0.10022 | 62.82 | **0.04416** |
| Ccl3 | 474.13 | **0.01009** | 2.37 | 0.50429 | 1335.16 | **0.01804** | 429.42 | **0.03928** |
| Ccl4 | 22.69 | **0.01615** | 2.24 | 0.43263 | 106.36 | 0.22715 | 19.87 | **0.02496** |
| Ccl5 | 4.32 | 0.49904 | 2.89 | 0.99685 | 16.05 | 0.24306 | 3.86 | 0.68902 |
| Ccl7 | 0.74 | 0.47253 | 2.88 | 0.10610 | 8.90 | 0.23316 | 1.34 | 0.23379 |
| Ccl8 | 0.89 | 0.96373 | 2.01 | 0.76543 | 9.11 | 0.28171 | 759.65 | 0.28559 |
| Ccr1 | 9.11 | 0.15381 | 1.29 | 0.90946 | 158.67 | 0.28515 | 10.96 | 0.07290 |
| Ccr2 | 28.16 | **0.03985** | 0.59 | 0.47096 | 430.09 | 0.28491 | 23.58 | **0.02874** |
| Ccr3 | 60.10 | **0.03229** | 1.38 | 0.93059 | 312.23 | 0.27465 | 41.80 | **0.04637** |

**Table S2.** *Cont.*

| Ccr4 | 192.49 | **0.01891** | 3.25 | 0.84918 | 3521.34 | 0.28421 | 83.22 | 0.10870 |
| --- | --- | --- | --- | --- | --- | --- | --- | --- |
| Ccr7 | 52.38 | 0.12078 | 0.60 | 0.38045 | 343.44 | 0.28548 | 14.45 | 0.11808 |
| Cd40 | 59.15 | 0.13549 | 1.64 | 0.65614 | 90.67 | 0.24942 | 57.39 | 0.08436 |
| Cd40lg | 52.94 | **0.04112** | 1.77 | 0.56741 | 1058.39 | 0.26061 | 50.11 | **0.01677** |
| Cebpb | 1.56 | 0.30369 | 3.51 | **0.04471** | 1.14 | 0.45276 | 0.60 | 0.50483 |
| Crp | 4.99 | 0.25188 | 0.51 | 0.54953 | 195.21 | 0.27480 | 19.70 | 0.05345 |
| Csf1 | 1.98 | **0.01157** | 2.12 | **0.00484** | 25.46 | 0.20771 | 2.70 | **0.04597** |
| Cxcl1 | 66.70 | **0.04105** | 2.16 | **0.03299** | 384.09 | 0.27292 | 36.27 | 0.09134 |
| Cxcl10 | 0.23 | 0.33800 | 0.52 | 0.29970 | 4.01 | 0.28666 | 0.37 | 0.39440 |
| Cxcl11 | 717.54 | 0.07050 | 5.63 | 0.35154 | 1191.86 | 0.48516 | 200.19 | **0.01247** |
| Cxcl2 | 22.69 | 0.14328 | 2.73 | **0.03217** | 135.49 | 0.13921 | 4.57 | **0.02457** |
| Cxcl3 | 4.105.24 | 0.16325 | 4.97 | 0.18663 | 26226.67 | 0.28559 | 10626.49 | 0.07072 |
| Cxcl5 | 1.117.67 | 0.16749 | 3.66 | 0.10879 | 1835.60 | 0.21627 | 330.09 | 0.28168 |
| Cxcl9 | 2.52 | 0.75743 | 1.04 | 0.39806 | 65.28 | 0.28567 | 37.71 | 0.09304 |
| Cxcr4 | 37.31 | 0.05733 | 0.80 | 0.49325 | 72.74 | 0.25313 | 30.28 | **0.02320** |
| Fasl | 23.94 | 0.09011 | 5.14 | **0.03603** | 635.68 | 0.17583 | 49.13 | **0.03387** |
| Flt3l | 2.58 | 0.28149 | 2.15 | 0.18083 | 28.55 | 0.23337 | 1.99 | 0.21542 |
| Fos | 15.91 | **0.00811** | 5.52 | 0.46421 | 37.81 | 0.13065 | 12.32 | **0.01765** |
| Hdac4 | 356.95 | **0.00113** | 1.20 | 0.16757 | 263.67 | 0.12715 | 268.02 | **0.00639** |
| IFN-γ | 120.76 | **0.01963** | 4.15 | **0.02184** | 400.43 | 0.15720 | 208.01 | 0.13203 |
| Il10 | 54.80 | **0.02424** | 2.84 | **0.03204** | 690.28 | 0.06185 | 33.25 | **0.01573** |
| Il10rb | 0.80 | 0.42746 | 2.86 | **0.01141** | 6.66 | 0.28562 | 0.66 | 0.35006 |
| Il18 | 0.04 | 0.08908 | 2.60 | **0.02003** | 0.55 | 0.37536 | 0.02 | 0.05487 |
| Il18rap | 278.21 | **0.05547** | 3.61 | 0.08153 | 1916.72 | 0.28550 | 166.23 | 0.07239 |
| Il1a | 578.07 | 0.11811 | 1.91 | 0.27238 | 1917.05 | 0.28558 | 251.11 | 0.09818 |
| Il1β | 96.37 | 0.09176 | 3.74 | **0.02111** | 218.86 | 0.26863 | 70.13 | 0.23807 |
| Il1f10 | 27.27 | **0.03741** | 6.89 | **0.02450** | 1314.85 | 0.26966 | 128.24 | 0.24554 |
| Il1r1 | 0.05 | 0.05582 | 2.04 | 0.16317 | 2.98 | 0.28545 | 0.37 | 0.47355 |
| Il1rap | 0.13 | **0.00005** | 1.40 | 0.13918 | 5.33 | 0.27407 | 0.74 | 0.62622 |
| Il1rn | 1.11 | 0.84867 | 3.24 | 0.18534 | 42.66 | 0.25076 | 4.22 | 0.14836 |

**Table S2.** *Cont.*

| Il22 | 21.18 | **0.00063** | 8.26 | **0.03113** | 1721.92 | 0.26975 | 144.16 | 0.14236 |
| --- | --- | --- | --- | --- | --- | --- | --- | --- |
| Il22ra2 | 144.01 | 0.17816 | 2.30 | 0.66765 | 5060.74 | **0.01959** | 117.87 | 0.26872 |
| Il23a | 442.00 | 0.14722 | 1.26 | **0.02134** | 1913.61 | **0.02649** | 244.57 | **0.02066** |
| Il23r | 124.32 | 0.09509 | 8.72 | **0.02330** | 3393.01 | 0.28478 | 183.28 | **0.00087** |
| Il6 | 1.183.50 | **0.03040** | 4.32 | **0.01248** | 7409.06 | 0.28555 | 819.36 | 0.05315 |
| Il6ra | 23.26 | 0.19008 | 2.18 | **0.01291** | 125.12 | 0.28360 | 13.42 | **0.01578** |
| Il7 | 151.64 | **0.03228** | 2.78 | 0.11752 | 733.56 | 0.28486 | 62.28 | **0.02760** |
| Il8ra | 22.41 | 0.15731 | 0.82 | 0.66114 | 144.46 | 0.18221 | 14.57 | 0.06679 |
| Il8rb | 8.41 | 0.21460 | 1.98 | 0.49962 | 509.94 | 0.27423 | 21.09 | 0.08296 |
| Il9 | 1.338.61 | **0.00121** | 9.01 | **0.04040** | 8251.12 | 0.18536 | 485.00 | 0.05672 |
| Itgb2 | 31.68 | **0.00206** | 2.01 | 0.84753 | 87.18 | 0.17790 | 19.48 | **0.00747** |
| Kng1 | 439.42 | **0.03010** | 2.05 | 0.91494 | 927.15 | 0.24341 | 210.56 | **0.01785** |
| Lta | 3.780.08 | **0.00059** | 2.22 | 0.77318 | 7038.77 | **0.02085** | 2945.16 | **0.01506** |
| Ltb | 112.03 | 0.14891 | 0.92 | 0.56214 | 2049.66 | 0.15165 | 34.72 | 0.27082 |
| Ly96 | 2.32 | 0.24028 | 2.59 | **0.03201** | 32.11 | 0.28202 | 0.57 | 0.44221 |
| Myd88 | 83.29 | 0.24474 | 1.52 | **0.01271** | 446.33 | 0.11206 | 64.35 | 0.28558 |
| Nfatc3 | 1.96 | 0.23314 | 1.65 | 0.16336 | 4.27 | 0.13602 | 1.11 | 0.34837 |
| Nfkb1 | 0.27 | 0.68756 | 2.19 | 0.09230 | 5.85 | 0.28571 | 0.42 | 0.95712 |
| Nos2 | 223.62 | 0.17769 | 2.59 | 0.21032 | 2161.25 | 0.21347 | 67.32 | 0.14014 |
| Nr3c1 | 0.01 | **0.01084** | 2.26 | **0.04965** | 0.34 | 0.41997 | 0.12 | 0.14695 |
| Ripk2 | 28.91 | **0.03903** | 1.93 | **0.01673** | 104.75 | 0.12437 | 24.47 | **0.01802** |
| Tirap | 14.48 | **0.01185** | 1.37 | 0.27102 | 26.21 | 0.14004 | 8.40 | **0.00099** |
| Tlr1 | 10.93 | 0.68972 | 1.07 | **0.01287** | 99.20 | 0.39791 | 6.23 | **0.02902** |
| Tlr2 | 1.46 | 0.31794 | 1.09 | 0.60997 | 13.86 | 0.25911 | 2.08 | **0.03310** |
| Tlr3 | 1.18 | 0.41258 | 1.06 | 0.84322 | 14.35 | 0.26521 | 1.27 | 0.33822 |
| Tlr4 | 0.41 | 0.24283 | 0.87 | 0.71187 | 1.09 | 0.34258 | 0.62 | 0.68007 |
| Tlr5 | 6.48 | 0.41573 | 1.45 | **0.01832** | 121.86 | 0.28505 | 8.70 | **0.01401** |
| Tlr6 | 146.63 | 0.34089 | 0.65 | 0.44187 | 944.13 | 0.28558 | 217.04 | **0.03197** |
| Tlr7 | 1.122.66 | 0.62935 | 1.28 | 0.08893 | 6164.53 | 0.48453 | 488.04 | 0.40022 |

**Table S2.** *Cont.*

| TNF-α | 363.40 | **0.02602** | 1.20 | **0.01840** | 2074.68 | **0.06697** | 83.80 | 0.05505 |
| --- | --- | --- | --- | --- | --- | --- | --- | --- |
| Tnfsf14 | 42.59 | **0.02631** | 1.67 | 0.63270 | 161.75 | 0.28486 | 26.35 | 0.12102 |
| Tollip | 0.81 | 0.85867 | 1.47 | 0.19308 | 2.82 | 0.28536 | 0.69 | 0.35820 |

© 2015 by the authors; licensee MDPI, Basel, Switzerland. This article is an open access article distributed under the terms and conditions of the Creative Commons Attribution license (http://creativecommons.org/licenses/by/4.0/).
